# DKC1 Overexpression Induces a More Aggressive Cellular Behavior and Increases Intrinsic Ribosomal Activity in Immortalized Mammary Gland Cells

**DOI:** 10.3390/cancers12123512

**Published:** 2020-11-25

**Authors:** Ania Naila Guerrieri, Federico Zacchini, Carmine Onofrillo, Sara Di Viggiano, Marianna Penzo, Alessio Ansuini, Ilaria Gandin, Yuko Nobe, Masato Taoka, Toshiaki Isobe, Davide Treré, Lorenzo Montanaro

**Affiliations:** 1 Department of Experimental, Diagnostic and Specialty Medicine, University of Bologna, Via Massarenti 9, 40138 Bologna, Italy; anianaila.guerrieri@studio.unibo.it (A.N.G.); federico.zacchini3@unibo.it (F.Z.); carmine.onofrillo@unimelb.edu.au (C.O.); sara.diviggiano@studio.unibo.it (S.D.V.); marianna.penzo@unibo.it (M.P.); davide.trere@unibo.it (D.T.); 2 Center for Applied Biomedical Research (CRBA), Alma Mater Studiorum, University of Bologna, Via Massarenti 9, 40138 Bologna, Italy; 3 Research Unit, AREA Science Park, Padriciano, 99, 34149 Trieste, Italy; alessio.ansuini@areasciencepark.it (A.A.); ilaria.gandin@areasciencepark.it (I.G.); 4 Department of Chemistry, Graduate School of Science and Engineering, Tokyo Metropolitan University, Minami-osawa 1-1, Hachioji-shi, Tokyo 192-0397, Japan; nobe-yuuko@tmu.ac.jp (Y.N.); mango@tmu.ac.jp (M.T.); isobe-toshiaki@tmu.ac.jp (T.I.); 5 Programma Dipartimentale di Medicina di Laboratorio, Azienda Ospedaliero-Universitaria di Bologna, Via Albertoni 15, 40138 Bologna, Italy

**Keywords:** dyskerin, DKC1, breast cancer, prognosis, snoRNA, rRNA, pseudouridylation

## Abstract

**Simple Summary:**

Dyskerin is a nucleolar protein involved in the modification and processing of ribosomal RNA and in the stabilization of the telomerase RNA component. In several human tumors, including breast cancer, dyskerin overexpression is found related to patients’ worse prognosis. Our aim was to study this phenomenon at the molecular and cellular levels. We firstly confirmed the correlation between high dyskerin expression with patients’ shorter survival. Then, through the generation of cellular models of increased dyskerin expression, we found that increasing dyskerin levels conferred a more aggressive phenotype and increased intrinsic ribosomal activity only in cells derived from normal breast epithelium. Our study provides evidence on the prognostic and bio-pathological relevance of the overexpression of dyskerin in breast carcinoma. A possible mechanistic explanation of the effects of dyskerin overexpression, involving specific ribosomal RNA modification and consequent increased ribosomal activity, is also provided.

**Abstract:**

Dyskerin is a nucleolar protein involved in the small nucleolar RNA (snoRNA)-guided pseudouridylation of specific uridines on ribosomal RNA (rRNA), and in the stabilization of the telomerase RNA component (hTR). Loss of function mutations in DKC1 causes X-linked dyskeratosis congenita, which is characterized by a failure of proliferating tissues and increased susceptibility to cancer. However, several tumors show dyskerin overexpression. We observed that patients with primary breast cancers with high dyskerin levels are more frequently characterized by shorter survival rates and positive lymph node status than those with tumors with a lower dyskerin expression. To functionally characterize the effects of high dyskerin expression, we generated stably overexpressing DKC1 models finding that increased dyskerin levels conferred a more aggressive cellular phenotype in untransformed immortalized MCF10A cells. Contextually, DKC1 overexpression led to an upregulation of some snoRNAs, including SNORA67 and a significantly increased U1445 modification on 18S rRNA, the known target of SNORA67. Lastly, we found that dyskerin overexpression strongly enhanced the synthetic activity of ribosomes increasing translational efficiency in MCF10A. Altogether, our results indicate that dyskerin may sustain the neoplastic phenotype from an early stage in breast cancer endowing ribosomes with an augmented translation efficiency.

## 1. Introduction

Dyskerin is a highly conserved 58 KDa nucleolar protein encoded by the DKC1 gene, mapped to chromosome Xq28 [1]. Dyskerin is involved in several cellular processes, the main two being RNA modification and telomerase complex stabilization. As for the RNA modification function, dyskerin is part of the pseudouridylation complex, with its catalytic activity necessary for isomerizing specific uridine residues to pseudouridines, mainly on ribosomal RNA (rRNA) and small nuclear RNA (snRNA). In this step, dyskerin is guided by a class of small nucleolar RNAs (snoRNAs), localized in the nucleolus or in Cajal bodies (scaRNAs [2]), which are provided with unique sequence features consisting of a two-hairpin and two single-stranded regions termed hairpin–hinge–hairpin–tail [3]. The hinge structure contains the so-called H box (consensus ANANNA) and the 3′ tail the ACA box (ACA); for this reason this class of RNAs is termed H/ACA box snoRNA. In this context, DKC1 together with three other structural proteins—NOP10, NHP2, and GAR1—and one guide snoRNA constitute the H/ACA small nucleolar ribonucleoproteins (snoRNPs) [4].

On the other hand, dyskerin is part of the telomerase complex in which it stabilizes the human telomerase RNA (hTR), characterized by the same H/ACA box structure as described above.

Germline DKC1 mutations cause X-linked dyskeratosis congenita (X-DC), an inherited syndrome characterized by a failure of proliferating tissues, such as bone marrow and skin, and increased susceptibility to cancer [5,6]. In X-DC, decreased levels of rRNAs pseudouridylation at specific sites [7,8] and reduced hTR stability resulting in dysregulated telomerase complex activity [9], are observed.

In several tumor types, such as breast [10], prostate [11], liver [12], and lung [13] cancers, dyskerin is often overexpressed, and this factor is frequently associated with an unfavorable prognosis. In particular, in a previous study, we demonstrated that tumors with higher dyskerin expression display an increased level of rRNA pseudouridylation together with higher hTR expression levels compared to tumors with a lower dyskerin expression [14]. Furthermore, patients with dyskerin-overexpressing tumors have a worse prognosis compared to patients with lower dyskerin levels [14].

Although several studies have demonstrated the role of dyskerin as a tumor suppressor [6,15,16,17,18], there are few studies in literature investigating the effects of dyskerin overexpression on the neoplastic phenotype. In this work we generated DKC1-overexpressing cell lines through a stable retroviral transduction approach, to assess the cellular and molecular effects of dyskerin overexpression. Our findings demonstrate that dyskerin overexpression confers a more aggressive phenotype in the immortalized mammary epithelial cells associated with a remodulation of H/ACA box snoRNAs expression, changes in uridine modification at a specific site, and an increased intrinsic ribosomal activity.

## 2. Results

### 2.1. DKC1 Overexpression Is Related to Worse Prognosis for Patients, and Confers a More Aggressive Neoplastic Features to Immortalized Mammary Gland Epithelial Cells In Vitro

**Figure 1:**
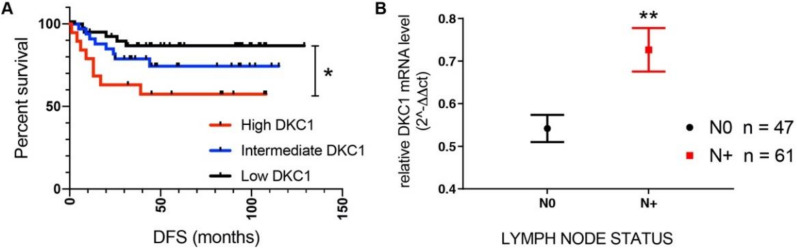
DKC1 overexpression is related to patients’ worse prognosis. (**A**) Disease free survival (DFS) of patients who had undergone surgery for primary breast carcinoma according to cancer cells DKC1 mRNA expression. Low DKC1 *n* = 54 mean expression value 0.39 ± 0.08 a.u.; Intermediate DKC1 *n* = 48 mean expression value 0.68 ± 0.10 a.u.; High DKC1 *n* = 22 mean expression value 1.25 ± 0.08 a.u. Data were analyzed by Mantel–Cox and Gehan–Breslow–Wilcoxon tests. * *p* < 0.05. (**B**) DKC1 mRNA expression and lymph node status in primary breast carcinomas. Data were analyzed by Mann-Whitney-test ** *p* < 0.01. The error bars indicate the SD.

In a previous study [14], we presented follow-up data from 62 patients with primary breast carcinoma in which patients with tumors expressing high DKC1 mRNA levels were characterized by a worse prognosis than patients with intermediate or low levels. We increased the patient number (170 in total, 124 with follow-up data) and updated follow-up information confirming the correlation between DKC1 expression and patients’ survival (Figure 1A). The association among DKC1 mRNA levels and patient prognosis was further confirmed in a larger series from the Pan-Cancer study (Breast Invasive Carcinoma TCGA PanCancer dataset available online [19]) (Figure S1). In addition, in our series we also found a significant correlation between DKC1 mRNA levels and lymph node status: tumors of patients with a lymph node status of N1 or higher (grouped N+) showed higher DKC1 mRNA levels than those of patients without nodal metastasis (N0) (Figure 1B). According to this observation DKC1 expression gradually increased from 0.54 ± 0.21 arbitrary units (a.u.) in the 47 N0 tumors, to 0.64 ± 0.34 a.u. in the 36 N1 tumors and finally to 0.84 ± 0.44 a.u. in the 25 tumors staged N2 or N3 (Mann-Whitney test *p* = 0.021). We also evaluated DKC1 mRNA expression in the cases from our series grouped according to their bio-profile classification. No significant differences in DKC1 mRNA expression among HER2 positive tumors, Luminal A, Luminal B, and triple negative tumors were observed (Kruskal–Wallis test *p* = 0.790). DKC1 mRNA-based classification turned out to be an independent variable (Cox regression analysis *p* = 0.047) in a multivariate survival analysis including bio-profile classification.

**Figure 2:**
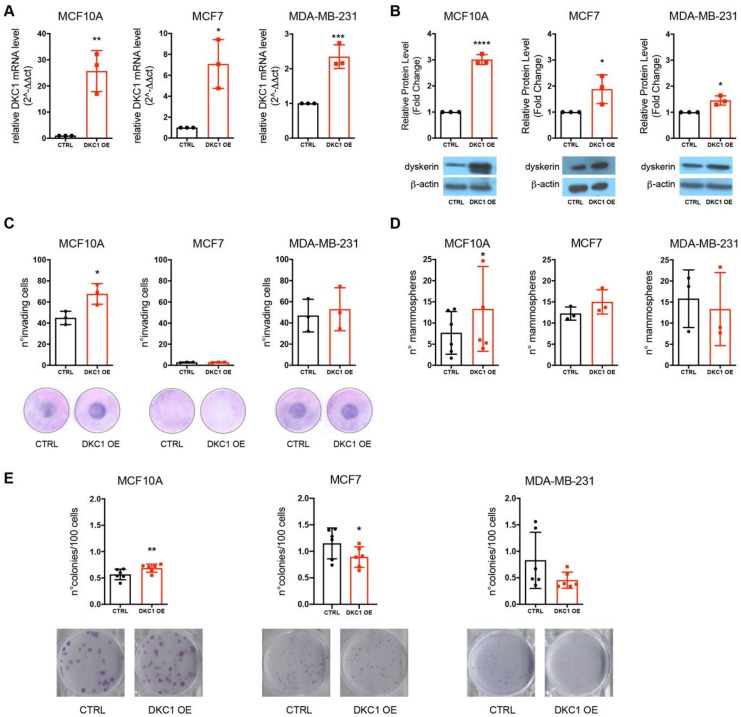
DKC1 overexpression confers a more aggressive cellular phenotype to immortalized mammary gland epithelial cells. (**A**) DKC1 mRNA expression evaluated by RT-qPCR. Results are shown as fold change respect to CTRL, set to 1 for all the three cell lines. (**B**) Dyskerin expression by Western blot analysis. Results are shown as fold change respect to CTRL, set to 1 for all the three cell lines. (**C**) Invasive potential assay through blind well assay chambers. For each filter sample five random fields were counted. Graphs show the total number of cells counted in each filter. (**D**) Stemness potential assay through mammospheres formation. Due to the intrinsic subjective nature of the experiment, we repeated it six times for MCF10A. (**E**) Clonogenic potential assay. At least *n* = 3 biological and technical replicates were performed for each experiment. All data were analyzed by paired Student’s *T*-test. * *p* < 0.05; ** *p* < 0.01; *** *p* < 0.001, **** *p* < 0.0001. The error bars indicate the SD.

To investigate the effects of dyskerin overexpression in tumors in vitro, we induced a stable DKC1 overexpression through the retroviral transduction of DKC1 cDNA in three cell lines with different basal dyskerin expression (Figure S2), thus recapitulating distinct levels of transformation in vitro: From lowest to highest, MCF10A (immortalized untransformed mammary epithelium cells), MCF7 (estrogen-positive invasive breast ductal carcinoma-derived cells), and MDA-MB-231 (triple negative invasive breast ductal carcinoma-derived cells). Cell lines were retrovirally transduced with an empty control vector (pMMLV[EXP]:IRES:Bsd, referred to as CTRL: Control) and a hDKC1 coding sequence (pMMLV[EXP]hDKC1[NM_001363.3]:IRES:Bsd, referred to as DKC1 OE: DKC1-overexpressing cells). After selection, we verified that dyskerin had been successfully overexpressed both at mRNA and protein levels for all three cell lines (Figure 2A,B). In general, the greatest increase in dyskerin overexpression had been achieved in MCF10A cells, which displayed the lowest dyskerin basal expression (Figure S2). We then performed in vitro assays to evaluate the effects of DKC1 overexpression on cellular invasive, stemness, and clonogenic potentials. In keeping with what was found in vivo, these results showed that an increasing DKC1 expression conferred a more aggressive phenotype, in terms of a greater number of invasive cells, number of mammospheres, and colonies comparable to that in control cells, only in MCF10A cells (Figure 2C–E).

To independently verify these observations, we further evaluated the effect of transient dyskerin overexpression on cell proliferation in the easy to transfect untransformed non-tumorigenic HEK293FT and osteosarcoma-derived U2OS cell lines. Obtained results indicate that in HEK293FT cells, which are derived from adenovirus type 5-immortalized human embryonic kidney cells [20]), DKC1 overexpression induced a significant increase in cell proliferation. Conversely, the overexpression of dyskerin did not elicit any effect on the proliferation of the osteosarcoma derived U2OS cells [21] (Figure S3).

### 2.2. DKC1 Overexpression Induced a Significant Increase in Telomerase RNA Component without Affecting Telomerase Activity

**Figure 3:**
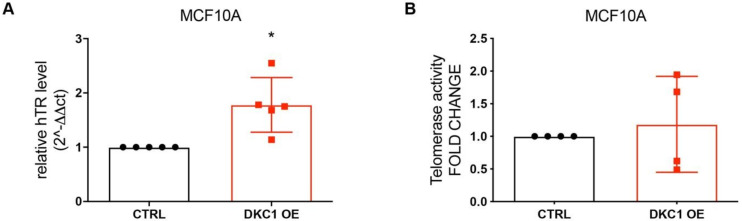
DKC1 overexpression leads to an increase in human telomerase RNA (hTR) levels without influencing telomerase complex activity. (**A**) Evaluation of hTR levels in Real-Time PCR. Data were analyzed by paired Student’s *T*-test: * *p* < 0.05. The error bars indicate the SD. (**B**) Telomerase activity assay. Statistical analyses have been conducted following protocol instructions. At least *n* = 3 biological and technical replicates were performed for each experiment.

We previously demonstrated that breast tumors with high dyskerin expression are also characterized by increased hTR levels [14]. For this reason, we verified whether dyskerin overexpression affected telomerase complex and activity. We evaluated hTR levels, finding a significant increase in MCF10A DKC1 OE cells with respect to the controls (Figure 3A). In parallel, no changes have been reported in MCF7, MDA-MB-231, and HEK293FT stable or transient dyskerin overexpression models (Figure S4). When we assessed telomerase activity, however, no significant differences were observed in MCF10A DKC1 OE and CTRL cells (Figure 3B). We previously reported that dyskerin mRNA levels are closely related to hTR and that DKC1 silencing in MCF7 cells leads to a drop in telomerase activity [14]. Indeed, telomerase activity is impaired when a fundamental component of the complex, such as hTR, is missing; on the other hand, an increase in the amount of other components of the telomerase complex that are not in limiting quantities, such as dyskerin, may not be sufficient to increase the activity of the whole complex.

### 2.3. DKC1 Overexpression Did Not Affect Global Pseudouridylation on rRNA, but Induced a Remodulation in snoRNAs Expression Levels

**Figure 4:**
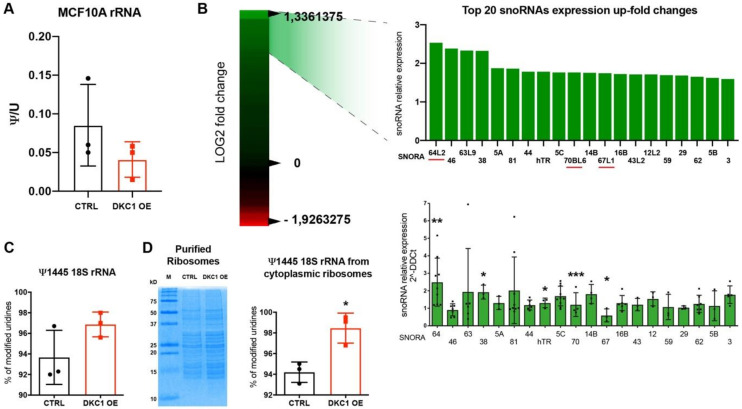
DKC1 overexpression did not influenced global rRNA pseudouridylation, but induced a remodulation in snoRNAs expression levels and a site-specific pseudouridylation increase in U1445 on 18S rRNA. (**A**) HPLC analysis of ribosomal RNA (rRNA) extracted from highly purified ribosomes. Results are shown as Ψ/U ratio. (**B** Left) Heat map of all H/ACA box small nucleolar RNA (snoRNAs) ordered by fold change (DKC1 OE/CTRL) LOG2. Green values are for more expressed snoRNAs, red values for less expressed. (**B** Right) Focus on top 20 up-regulated snoRNAs in MCF10A Dyskerin overexpressing cells from Arraystar expression array analyses (up). snoRNAs expression validated by RT-qPCR (down). Data were analyzed by paired Student’s *T*-test * *p* < 0.05; ** *p* < 0.01; *** *p* < 0.001. The error bars indicate the SD. (**C**) LC-MS analyses of Ψ1445 on 18S rRNA and (**D** right) on RNA from cytoplasmic ribosomes. Results are shown as percentage of modified uridines and were calculated from the peak area. Data were analyzed by unpaired Student’s *T*-test * *p* < 0.05. The error bars indicate the SD. (**D** left) Coomassie stain of a PAGE of proteins from the purified ribosomes. Note that following the purification procedure only bands of sizes corresponding to ribosomal proteins were detected. At least *n* = 3 biological and technical replicates were performed for each experiment.

In order to investigate the effects of dyskerin overexpression on pseudouridylation, we evaluated the global pseudouridylation level of total rRNA by HPLC analysis. To make sure that the analyzed rRNA was indeed completely mature, we extracted it directly from highly purified cytoplasmic ribosomes. The results obtained did not show any significant change in global pseudouridylation after dyskerin overexpression in MCF10A (Figure 4A). Similar results were observed also in MCF7 and in MDA-MB-231 cells (Figure S5). However, since there are nearly one hundred pseudouridylation sites on human rRNA [22], this result did not rule out a possible effect on the modification in specific sites. To understand whether a specific modification site may be affected, we tested the impact of dyskerin overexpression on the abundance of each single snoRNA through a comprehensive expression array, which analyzed 359 H/ACA and C/D box snoRNAs. We focused only on the former, which guide dyskerin and the pseudouridylation complex during its activity. The analysis showed that in MCF10A cells, DKC1 overexpression leads to a remodulation of snoRNAs abundance, with upregulation being the most frequent event. We decided to focus on the top 20 up-regulated snoRNAs (Fold Change from 1.5 to 2.5) and we were able to confirm, by Real-Time qPCR validation, a significant upregulation in four snoRNAs: SNORA64, SNORA70, SNORA67, and SNORA38 (Figure 4B). Three of these snoRNAs have known target uridines on rRNAs: SNORA64 targets U1692 on 18S rRNA, SNORA70 targets U4975 on 28S rRNA, and SNORA67 targets U1445 on 18S rRNA. To quantitatively assess the specific level of modification in these sites we performed a LC-MS (SILNAS-MS based quantitation [8]) analysis on MCF10A DKC1 OE and CTRL cells. For the three specific sites the analysis was initially performed on total cellular rRNA. While no changes were observed for U1692 on 18S rRNA and U4975 on 28S rRNA (Figure S6), an increasing trend in Ψ1445 on 18S rRNA was found in DKC1 OE cells (shown in Figure 4C). To investigate the modification of U1445 more in depth, we then performed the same kind of analysis on rRNA extracted from highly purified cytoplasmic ribosomes. Importantly, this analysis highlighted a significant difference in U1445 modification after DKC1 overexpression. Indeed, in this context of completely matured and terminally processed rRNAs, we found a significantly increased amount of Ψ1445, indicating that, in MCF10A DKC1-overexpressing cells, ribosomes are significantly more pseudouridylated at this specific site (Figure 4D). Interestingly, in MCF7 and MDA-MB-231 cells, where we did not observe any clear effect of DKC1 overexpression on cellular behavior, we also did not detect any significant effect neither on SNORA67 nor in SNORA70 and SNORA64 levels (Figure S7). In addition, the analysis of U1445 in ribosomes from MCF7 cells showed that this specific site is found fully modified in this cell line (Figure S8).

### 2.4. DKC1 Overexpression Increases Translation Efficiency of Ribosomes in MCF10A Cells

**Figure 5:**
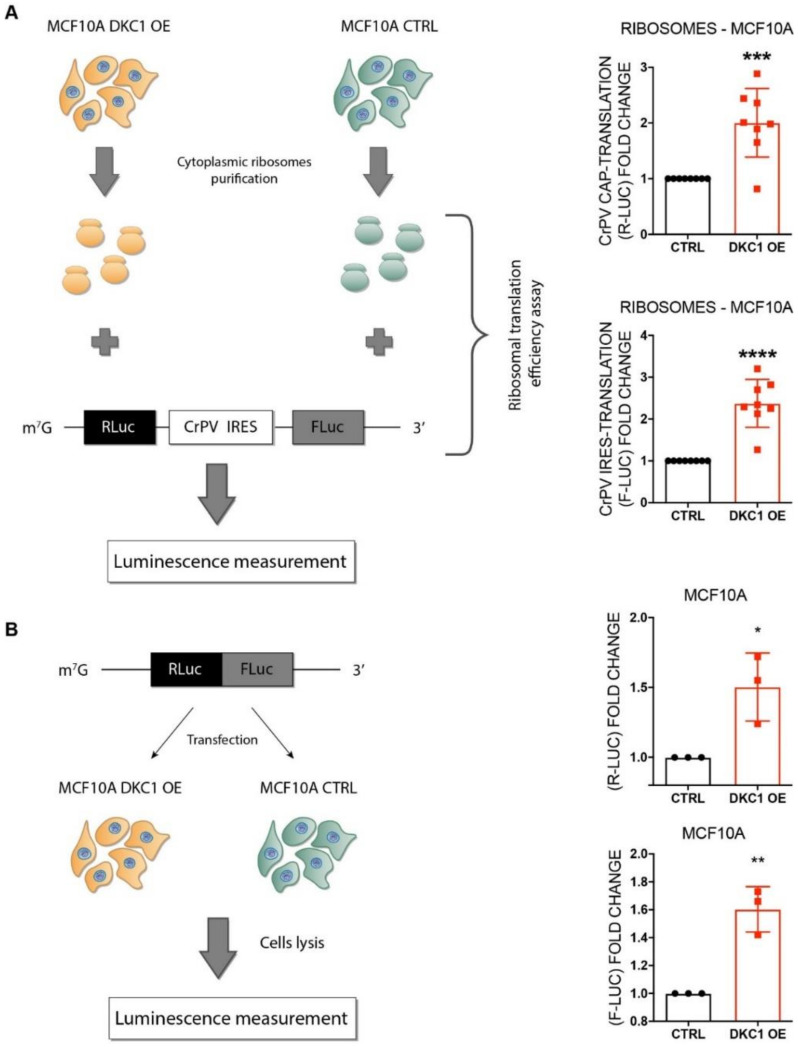
DKC1 overexpression provides ribosomes with increased translational efficiency in MCF10A cells. (**A**) mRNA translation efficiency assay conducted on highly purified ribosomes extracted from MCF10A DKC1 overexpressing cells and control cells. (**B**) mRNA translation efficiency assay on MCF10A DKC1 overexpressing and control cells. At least *n* = 3 biological replicates were performed, testing all sample in duplicate. Data were analyzed by unpaired or paired Student’s *T*-test as appropriate. * *p* < 0.05; ** *p* < 0.01; *** *p* < 0.001; **** *p* < 0.0001. The error bars indicate the SD.

To further investigate the impact of the observed U1445 modification on ribosome function, we purified ribosomes from MCF10A DKC1 OE and CTRL cells, and assessed their intrinsic translation efficiency in a reconstituted cell-free system [23]. We challenged purified ribosomes with a bicistronic R-LUC-IRES-F-LUC reporter vector to test both cap-dependent and -independent (IRES mediated) translation [23]. The bicistronic construct harbored the cricket paralysis virus (CrPV) IRES element in between the two cistrons. This specific IRES element is known to mediate translation initiation in the absence of cellular initiation factors, making it possible to more directly assess the contribution of intrinsic ribosomal features in cap-independent initiation [24]. In this kind of assay, the measured luminescence is proportional to cellular translational activity. Interestingly, we found that ribosomes purified from dyskerin-overexpressing cells were significantly more efficient in the translation of the reporter mRNA, independently from the translation initiation mode (cap- or IRES-mediated translation) (Figure 5A).

We then wondered whether the increase in intrinsic ribosomal translation efficiency affected the overall cellular translation efficiency. To address this point, an in vitro cell-based assay was performed; our MCF10A DKC1 OE and CTRL cells were transfected with a monocistronic mRNA encoding a chimeric luciferase endowed with both *Firefly* Luciferase (F-LUC) and *Renilla* Luciferase (R-LUC) activities. These experiments showed that in a cellular system as well as in the cell-free system, dyskerin overexpression induced a significant increase in reporter activity, suggesting an augmented translation efficiency (Figure 5B). To confirm these findings, we reduced DKC1 mRNA levels in MDA-MB-231 cells (which are characterized by high basal dyskerin expression) (Figure S9A). According to our observations in MCF10A cells, DKC1 mRNA depletion in MDA-MB-231 cells led to a reduction of SNORA64, SNORA67, and SNORA70 (Figure S9B) and to a decrease of the translational efficiency (Figure S9C).

## 3. Discussion

This work confirmed that patients with higher dyskerin expression are characterized by a worse prognosis than patients with lower DKC1 levels. In addition, our study showed that tumors characterized by high dyskerin expression metastasize more frequently to lymph nodes than other tumors. Consistently, in untransformed mammary epithelial cells, MCF10A, increased dyskerin expression conferred a more aggressive cellular features which include increased invasive, staminal and clonogenic potentials. These experimental findings well fit with the observations on patients’ survival and nodal status. Of all the tested cellular models, DKC1 overexpression induced an increase in hTR only in MCf10A cells. However, even in these cells, this was not sufficient to increase telomerase activity. On the other side, in MCF10A cells, DKC1 overexpression led to a significant increase in the levels of selected H/ACA box snoRNAs including SNORA67. This was associated with an increase in U1445 site-specific pseudouridylation in 18S rRNA of mature cytoplasmic ribosomes. These results allow us to hypothesize that the modification in this specific residue might be involved in the intrinsic activity of ribosomes. Recently, the study of Abeyrathne et al. presented high-resolution Cryo-EM structures of the 80S ribosome from S. Cerevisae bound with eEF2-GTP. Their model suggests that the 80S ribosome goes through five different conformational changes during translocation. Specifically, between structures II and V, the sliding of eEF2 implies that positively charged ribosomal domains hop from helix 33 to helix 34 (h34) precisely at the U1445 site [25]. Thus, U1445 is involved in the ribosome structural changes during the translation elongation phase, and so its site-specific pseudouridylation may possibly affect elongation efficiency. Therefore, we analyzed the impact of increased dyskerin levels in translation and found that ribosomes from dyskerin-overexpressing cells are significantly more efficient in translation than their controls, regardless of the translation initiation mode (cap or IRES translation) compared to the control ribosomes.

Altogether, our findings and the above-reported considerations suggest that dyskerin may play a direct role in supporting the neoplastic transformation of mammary epithelium being consistent with a model in which variable levels of DKC1-mediated pseudouridylation of rRNA nucleotides, such as U1445 on 18S rRNA, play an important role in regulating mRNA translation. In principle, such a surge in protein synthesis may, on the one hand, support the increased biomass demand of cancer cells and, on the other, also post-transcriptionally modify the expression of a specific subset of mRNAs. Further studies, in particular with other different mammary epithelium derived cellular models in addition to MCF10A, are needed to characterize these effects in detail; however, these results could explain the link between DKC1 expression and patients’ specific prognoses.

Over the years, several studies have demonstrated the active role of protein synthesis and ribosomal biogenesis in the neoplastic transformation process [26,27,28,29,30]. Highly proliferating neoplastic cells, in fact, require a significantly increased protein synthesis to meet their biosynthetic demands. On the other hand, there is increasing evidence in literature that snoRNAs may themselves contribute to malignancy in specific human cancer types, such as breast cancer [31,32,33]. Our results suggest that the upregulation of specific snoRNAs in human tumors may have an impact on the change of their targets on rRNA, thus modifying ribosomal activity and ultimately contributing to an increased cellular protein synthesis capability.

Lastly, Alawi et al. demonstrated that dyskerin is a direct and conserved target of MYC, linking the aggressive phenotype of tumors that show an upregulation of both proteins to the well-established oncogenic role of MYC [34,35,36]. Our study, however, suggests that in breast cancer some specific effects must be ascribed directly to the increased dyskerin expression, even independently from MYC. Furthermore, the same authors showed that dyskerin up-regulation is linked to an increase in cellular proliferation independently of TERT and telomerase activity [34,35]. These findings, in concert with our results, demonstrate that the role of dyskerin in tumorigenesis is not related with its function in the telomerase complex. In addition, considering that dyskerin activity may be pharmacologically inhibited [15], our work also provides a rationale for targeting this enzyme with therapeutic intents in human cancer.

## 4. Materials and Methods

### 4.1. Cell Culture and Generation of DKC1 Overexpression Models

Immortalized untransformed MCF10A mammary epithelial cells were cultured in DMEM 1 g/L glucose supplemented with 250 U/L of insulin, 0.5 μg/mL of hydrocortisone, 10 ng/mL of epidermal growth factor, 20% fetal bovine serum (FBS), 2 mM L-glutamine, 100 U/mL penicillin, and 1 mg/mL Streptomycin, and 4 μg/mL blasticidin. Estrogen-positive invasive breast ductal carcinoma-derived MCF7 cells were cultured in RPMI 1640 supplemented with 10% FBS, 2 mM l-glutamine, 100 U/mL penicillin, 1 mg/mL streptomycin, and 2.5 μg/mL blasticidin. Triple-negative invasive breast ductal carcinoma-derived MDA-MB-231 cells were cultured in DMEM 4.5 g/L glucose supplemented with 10% FBS, 2 mM L-glutamine, 100 U/mL penicillin, 1 mg/mL streptomycin, and 7 μg/mL blasticidin.

All cell lines were obtained from the American Type Culture Collection (ATCC, Manassas, VA, USA).

To generate stable DKC1-overexpression cell models, all cell lines were infected with Moloney Murine Leukemia Virus (MoMLV) containing either control plasmid {pMMLV [EXP]-Bsd(IRES:Bsd), referred to as CTRL)} or hDKC1 sequence {pMMLV [EXP]hDKC1 [NM_001363.3]:IRES:Bsd, referred to as DKC1 OE}. After the infection cells were selected with blasticidin (8 μg/mL MCF10A; 5 μg/mL MCF7; 14 μg/mL MDA-MB-231) for at least 10 days, DKC1 overexpression was verified at both the mRNA (Real-Time qPCR) and protein level (Western blot). The so obtained cell lines derive from more than one independent retroviral transduction and, in order to minimize the impact of a low number of selected clones, cellular populations deriving from each single transduction were joined during the antibiotic selection. All the experiments have been conducted at early passages to avoid late clonal selection or unpredictable adaptation events.

All reagents and supplements were supplied by Sigma-Aldrich, St. Louis, MO, USA.

All cells were cultured in a monolayer at 37 °C and 5% CO_2_ in a humidified incubator.

### 4.2. RNA Extraction and Real-Time RT-PCR

RNAs were extracted using PureZOL™ RNA Isolation Reagent (Bio-Rad Laboratories, Irvine, CA, USA), following the manufacturer’s specifications. cDNA was synthesized starting from 500 ng of RNA (or 10 ng for hTR level evaluation) using an iScript™ cDNA Synthesis Kit (Bio-Rad Laboratories) following protocol instructions.

Real-time qPCR analyses were conducted with a CFX96™ Real-Time detection System (Bio-Rad Laboratories). A semi-quantitative Taqman approach (SsoAdvanced Universal Probes Supermix, Bio-Rad Laboratories) was used to evaluate the expression of DKC1 and β-glucuronidase as endogenous control (Applied Biosystems, Bedford, MA, USA; Hs 00154737_mL and 4326320E, respectively). Regarding hTR level evaluation, a Taqman approach was used as described elsewhere [37]. A SYBR green approach (SsoAdvanced™ Universal SYBR^®^ Green Supermix; Bio-Rad Laboratories) was used to measure relative mRNAs levels of snoRNAs: SNORA64 For 5′-GTGTGACTTTCGTAACGGGGA-3′ Rev 5′-TTGCACCCCTCAAGGAAAGAG-3′; SNORA63 For 5′-AGCAGGATTCAGACTACAATATAGC-3′ Rev 5′-GCTACAGGAGAATAGCAGACAG-3′; SNORA81 For 5′-AATTGCAGACACTAGGACCAT-3′ Rev 5′-GGACATTGGACATTAAGAAAGAGG-3′; SNORA43 For 5′-GGGCAAAGAGAAAGTGGCGA-3′ Rev 5′-GGCCATAAACCATTCTCAGTGC-3′; SNORA46 For 5′-TCTTGGTTACGCTGTAGTGC-3′ Rev 5′-ACTCTATACAGCAACAGCAGAAT-3′; SNORA5A For 5′-AGCCGTGTCAAATTCAGTACC-3′ Rev 5′-GCCCATGAGTCACAGTGTTT-3′; SNORA44 For 5′-CATGCAAGAGCAACCTGGAA-3′ Rev 5′-TATAGGAAAGCTGAGTGGCAG-3′; SNORA5C For 5′-AGTGCCCGTTTCTGTCATAGC-3′ Rev 5′-CAAACTTATCCCCAGGTCCCA-3′; SNORA70 For 5′-CCGACTGAGTTCCTTTCCACA-3′ Rev 5′-AGGCTGCGTACACTACCAAG-3′; SNORA5B For 5′-AGCCATGTCAAATTCAGTGCCT-3′ Rev 5′-ACTGTTTCTGTGGCAGTCTTCT-3′; SNORA12 For 5′-CAAATGGGCCTAACTCTGCC-3′ Rev 5′-TCTCTGATGCAGGAAAGGCT-3′; SNORA38 For 5′-GTGTCTGTGGTTCCCTGTCTT-3′ Rev 5′-GGCCTCAAAGTTTCCCAAATCC-3′; SNORA16B For 5′-GCTCCAGGTGCTTCCATGTAG-3′ Rev 5′-TCACCATCAAGGAAAACTGTCACT-3′; SNORA59 For 5′-GTATGTTCACGGGGCGATGC-3′ Rev 5′-TCTACGGGTAACTGAGGCAC-3′; SNORA29 For 5′-CATTTGACTACCACATTTTCTCCTA-3′ Rev 5′-TCCCTCTTCAGATCATGGCAAG-3′; SNORA62 For 5′-GGAGTTGAGGCTACTGACTGG-3′ Rev 5′-AGCGAAAACTTGCCCCTCAT-3′; SNORA3 For 5′-AGTCACGCTTGGGTATCGG-3′ Rev 5′-AGCCAGTGAATAAGGTCAGCA-3′.

As for snoRNAs 64L2, 63L9, 70BL6, 67L1, 43L2, and 12L2, since they have no known target RNA, we decided to validate the snoRNAs levels with homology in the pseudouridylation pocket sequence or whole sequence, hypothesizing a correspondence in the modified uridines.

### 4.3. Whole Cell Protein Extraction and Western Blot Analysis

Whole cell protein extraction was performed in a lysis buffer (KH2PO4 0.1 M pH 7.5, NP-40 1%, additioned with Complete protease inhibitor cocktail (Sigma-Aldrich) and 0.1 mM β-glycerolphosphate) for 20 min and cleared by centrifugation at 14,000 RCF for 20 min. Protein extract was quantified spectrophotometrically via the Bio-Rad Protein Assay (Bio-Rad Laboratories). The same amount of proteins was separated in Laemmli loading Dye (2% SDS; 8% glycerol; 62.5 mM TRIS HCL PH 6.8; 0.005% bromophenol blue and 2% β-mercaptoethanol) by SDS PAGE in a polyacrylamide gel (TGX Stain-Free™ FastCast™ Acrylamide Solutions, Bio-Rad Laboratories) in Running Buffer (2.5 mM Tris, 19.2 mM Glycine and 0.1% SDS) at constant 200 V for about 30 min. Proteins were then transferred onto a PVDF membrane (Amersham Hybond P 0.45 PVDF, GE Healthcare Life Sciences now Cytiva, Marlborough, MA, USA) with Transfer Buffer (2.5 mM Tris, 19.2 mM Glycine, 20% MetOH) for 2 hrs. Dyskerin antibody was purchased from Santa Cruz Biotechnology (Dallas, TX, USA) (H-300 sc-48794); β-actin antibody was purchased from Sigma-Aldrich (clone AC-74 No. A2228).

### 4.4. Cell Invasive, Clonogenic, and Stemness Potentials Assays

These assays were performed as described in detail in Galbiati et al. [38]. In particular, for the colonies formation assays 100 cells for MCF10A CTRL/DKC1 OE and MDA-MB-231 CTRL/DKC1 OE, or 250 cells for MCF7 CTRL/DKC1 OE, respectively, were seeded in a single 6-well plate and analyses were conducted as described in Galbiati et al. [38]. The experimental condition of the invasive potential assay of MCF7 DKC1-OE and CTRL cells were the same as MDA-MB-231 cells detailed in Galbiati et al. [38].

### 4.5. Telomerase Activity Assay

Telomerase activity assay was performed following manufacturer’s instructions (S7710—TRAPEZE RT, Sigma Aldrich). In brief, 10^5^ cells were lysed with 200 µL of CHAPS lysis buffer and 2 µL of each lysate were loaded for the Real-Time PCR analysis. Positive/negative controls and standard curve were loaded following protocol instructions. Platinum™ Taq DNA Polymerase (Invitrogen, Thermo Fisher Scientific, Waltham, MA, USA) was used as an antibody-mediated hot-start Taq polymerase for PCR reaction. Results were analyzed following protocol’s instruction.

### 4.6. Ribosome Purification

Human ribosomes from MCF10A CTRL/DKC1 OE were purified as described elsewhere [23]. In brief, cells were lysed with a lysis buffer, which enables to isolate the cytoplasmic fraction from the nuclei and mitochondria. After a short 10-min incubation at 37 °C, which permits ribosomes to finish translation and become detached from the mRNAs they were translating, up to 500 μL of the cytoplasmic lysate is loaded onto discontinuous sucrose gradient and ultracentrifuged for 15 h. The resulting pellet is dissolved in a suitable amount of a storage solution (10 mM Tris HCl pH 7.5, 2 mM magnesium acetate, and 100 mM ammonium acetate in RNase-free water) and ribosomes were quantified following the protocol indications described in Penzo et al. [23].

### 4.7. In Vitro Translation Assays

For whole cell system in vitro translation assays, 2 × 10^5^ cells (MCF10A DKC1 OE and CTRL) were seeded in 6-well plates. The day after, cells were transfected with 400 ng of the reporter monocistronic transcript pR-LUC-F-LUC (a kind gift from Kim De Keersmaecker, Department of Oncology, Laboratory for Disease Mechanisms in Cancer, KU Leuven, Belgium) using Lipofectamine RNAiMAX Transfection Reagent (Thermo Fisher Scientific, Waltham, MA, USA), following protocol instructions. After 5 h, cells were lysed with 500 μL of Passive Lysis Buffer 5× and luminescence was measured following the instructions of the Dual-Luciferase^®^ Reporter Assay System (Promega Madison, WI, USA). For in vitro IRES-mediated translation assays with highly purified ribosomes, we used the pR-CrPV-IRES-F plasmid (a kind gift from Dr. Davide Ruggero, Department of Urology, University of California, San Francisco, CA, USA) [39].

Capped mRNAs were transcribed from linearized plasmids using an AmpliCap-Max T7 High Yield Message Maker kit (CellScript, Madison, WI, USA), following the supplier’s instructions. mRNA translation efficiency assay of highly purified ribosomes was performed as described in Penzo et al. [23].

### 4.8. Global Pseudouridylation Quantification

The rRNA global pseudouridylation was evaluated through HPLC analysis as previously described in Montanaro et al. [14].

### 4.9. SnoRNAs Expression Array

SnoRNAs expression analysis was performed by Arraystar company using the nrStar™ Human snoRNA PCR Array which contains 359 snoRNAs, 7 snoRNA target snRNAs, and 4 snoRNP complex members. 2–5 µg of total RNA from MCF10A DKC1 OE/CTRL were shipped to Arraystar Inc. Experiment and data analyses were performed by Arraystar Inc (Rockville, MD, USA).

### 4.10. SILNAS LC/MS Based Quantitation of Ψs

The SILNAS-based quantitation of the stoichiometry of Ψs was performed as described in Taoka et al. [8].

### 4.11. Patients’ Material

One hundred and seventy breast carcinomas were selected from a series of consecutive patients who underwent surgical resection for primary breast carcinoma at the Surgical Department of the University of Bologna on the sole basis of frozen tissue availability for DKC1 mRNA expression determination. Part of the cases were obtained from a previous study [14] while additional samples were collected after 2011. Surrogate bio-profile classification of the cases on the basis of histological results was performed according to St. Gallen 2017 consensus [40] as in De Nicola et al. [41]. Informed consent was obtained from all individual participants included in the study. All procedures performed in studies involving human participants were in accordance with the ethical standards of the Institutional Research Committee (no. 75/2011/U/Tess approved on 19 July 2011 and 132/2015/U/Tess approved on 13 October 2015 by the Policlinico S. Orsola–Malpighi Ethical Review Board, Bologna, Italy), 1964 Helsinki Declaration as subsequently amended, or any comparable ethical standards.

### 4.12. Statistical Analyses

Survival analyses were performed according to Kaplan-Meier and Cox proportional hazard models. For each experiment, at least three biological and technical replicates were performed. Data were analyzed using SPSS program package Version 25 or GraphPad Prism Version 8. For statistical analyses, paired or unpaired t-test, Mann-Whitney and Kruskal–Wallis test were applied where appropriate. Values for *p* less than 0.05 were regarded as statistically significant. Details of the specific statistical analysis are described in figures’ captions. Statistical analyses regarding Supplementary Results are provided in the Supplementary Materials section.

## 5. Conclusions

Our study provides strong evidence on the prognostic and bio-pathological relevance of the overexpression of the human pseudouridine synthase dyskerin in breast carcinoma. Furthermore, a possible mechanistic explanation of the effects of dyskerin overexpression, involving rRNA modification at a specific site and consequent increased ribosomal activity, is provided. Further studies with other untransformed immortalized mammary epithelium-derived cellular models are needed to confirm the direct connection between dyskerin overexpression, more aggressive cellular phenotype and patients’ worse prognosis.

## Supplementary Materials

The following are available online at https://www.mdpi.com/2072-6694/12/12/3512/s1, Figure S1: The Pan-Cancer Clinical data Resource (hereinafter “TCGA cohort”) was analyzed to replicate our findings in the breast cancer series, Figure S2: Basal dyskerin expression in parental MCF10A, MCF7 and MDA-MB-231 evaluated by Western Blot, Figure S3: Evaluation of the transient DKC1 overexpression effect in the easy to transfect non tumorigenic untrasformed HEK293FT and in the osteosarcoma U2OS cell lines, Figure S4: Evaluation of hTR levels by Real-Time qPCR in stable DKC1 overexpressing MCF7 (**A**) and MDA-MB-231 (**B**) cells and in transient DKC1 overexpressing HEK293FT (**C**) cells, Figure S5: HPLC analyses of rRNA extracted from highly purified ribosomes from MCF7 and MDA-MB-231 DKC1 OE and CTRL cells. Results are shown as Ψ/U ratio, Figure S6: LC-MS analyses on Ψ1692 18S rRNA (**A**) and Ψ4975 28S rRNA (**B**) performed on MCF10A total cellular RNA, Figure S7: SNORA67, SNORA70 and SNORA64 expression evaluated by RT-qPCR in (**A**) MCF7 and (**B**) MDA-MB-231 stable dyskerin overexpression models, Figure S8: LC/MS analyses of Ψ1445 on 18S rRNA in parental MCF7 cell lines, Figure S9: Evaluation of the transient DKC1 knockdown (KD) effects in MDA-MB-231 cells. (**A**) DKC1 mRNA expression evaluated by RT-qPCR. Results are shown as fold change respect to CTRL, set to (**B**) SNORA64, SNORA70 and SNORA67 expression evaluated by RT-qPCR in MDA-MB-231 transient dyskerin KD models. (**C**) mRNA translation efficiency assay on MDA-MB-231 DKC1 KD and control cells.
