# Supplementary material for: DKC1 Overexpression Induces a More Aggressive Cellular Behavior and Increases Intrinsic Ribosomal Activity in Immortalized Mammary Gland Cells
Source: Cancers (Basel). 2020 Nov 25;12(12):3512. doi: 10.3390/cancers12123512 (PMC7760958; doi:10.3390/cancers12123512)
Supplement: Supplementary file 1 [file cancers-12-03512-s001.pdf]

Supplementary Materials:

DKC1 Overexpression Induces a More Aggressive Cellular Behavior and increases Intrinsic Ribosomal Activity in Immortalized Mammary Gland Cells

Ania Naila Guerrieri, Federico Zacchini, Carmine Onofrillo, Sara Di Viggiano, Marianna Penzo, Alessio Ansuini, Ilaria Gandin, Yuko Nobe, Masato Taoka, Toshiaki Isobe, Davide Treré and Lorenzo Montanaro

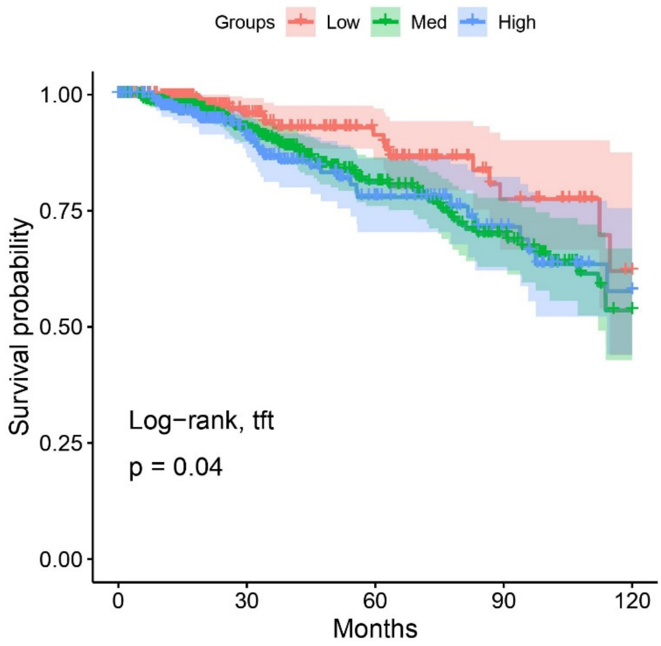

Figure S1. The Pan-Cancer Clinical data Resource (hereinafter "TCGA cohort") was analyzed to replicate our findings in the breast cancer series**.** Data were extracted from the public database cBioPortal which provided both DKC1 mRNA expression level (RSEM batch normalized expression values) and clinical information (cancer type, overall survival, etc.). The query was implemented through the R *package cgdsr* and the specific formulation is reported in Supplementary Materials. After restricting the analysis to invasive lobular and ductal carcinomas, removing one subject for missing data and two outliers based on expression levels, the cohort size was n = 967 (age 58 ± 13, mean ± SD). Survival analysis showed a significant trend (log-rank test for trend, p = 0.040) in DKC1 mRNA expression groups. Cox regression analysis showed that a 10% increase is associated with a hazard ratio of 1.04 (p = 0.025).

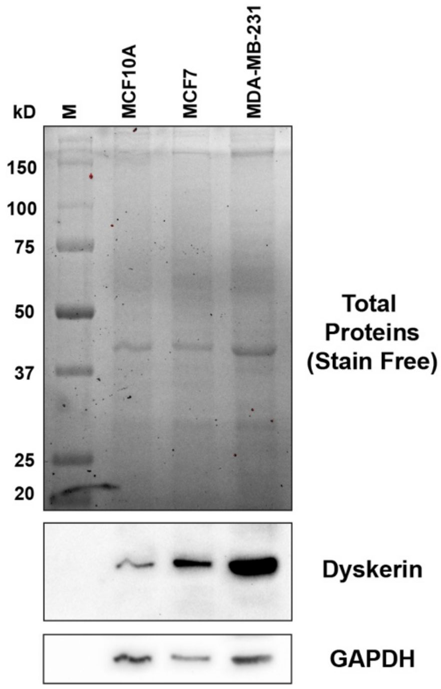

Figure S2. Basal dyskerin expression in parental MCF10A, MCF7 and MDA-MB-231 evaluated by Western Blot.

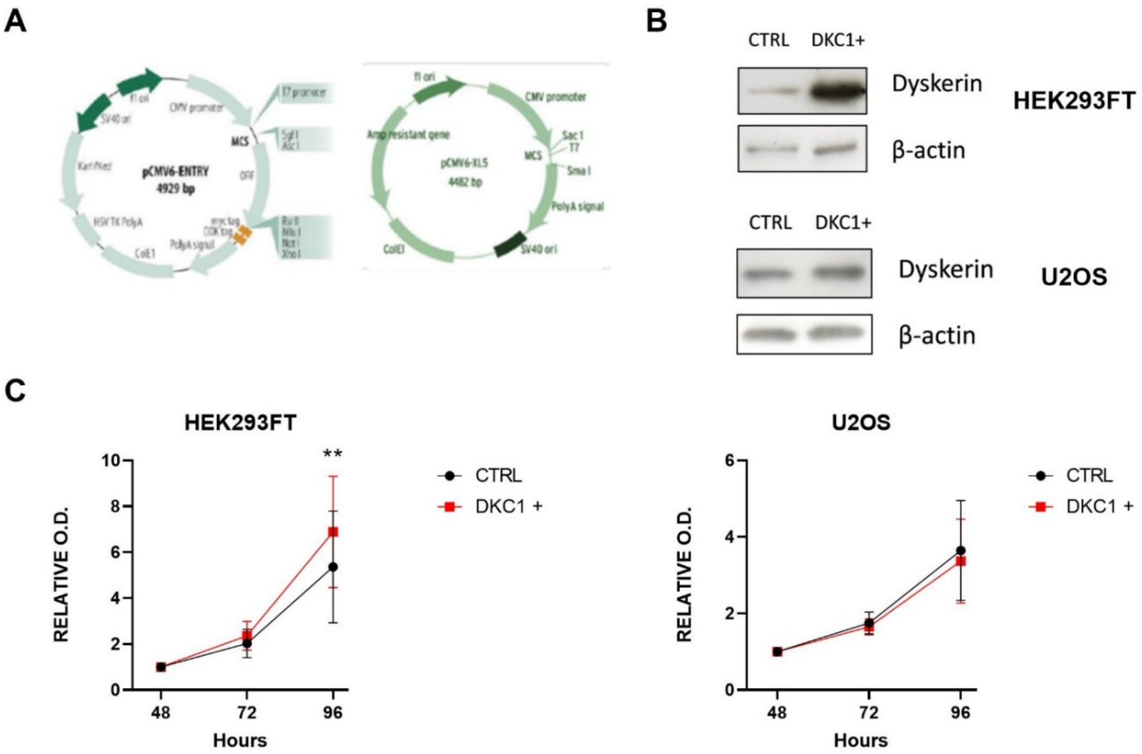

Figure S3. Evaluation of the transient DKC1 overexpression effect in the easy to transfect non tumorigenic untrasformed HEK293FT and in the osteosarcoma U2OS cell lines. (**A**) Maps of pCMV6-ENTRY (control) and pCMV-XL5-hDKC1 (DKC1+) plasmids (Origene). (**B**) Western blot analysis of dyskerin expression in HEK293FT and U2OS cells after 72 h from transfection. Overexpression levels decrease after 96- and 120-h post-transfection. (**C**) Growth curve of HEK293FT and U2OS DKC1+ and CTRL cells. Three biological and six technical replicates were performed. Two-way ANOVA Multiple Comparison has been used for statistical analyses of growth curve experiments. Error bars indicate SE.

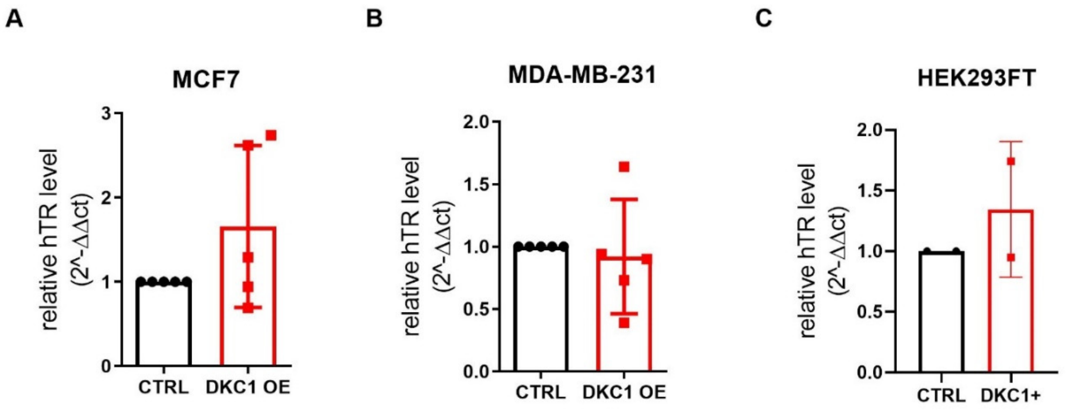

Figure S4. Evaluation of hTR levels by Real-Time qPCR in stable DKC1 overexpressing MCF7 (**A**) and MDA-MB-231 (**B**) cells and in transient DKC1 overexpressing HEK293FT (**C**) cells. Results are shown as fold change respect to controls set to 1. Experiments have been performed in technical triplicate of three biological replicates. No significant statistical differences have been found.

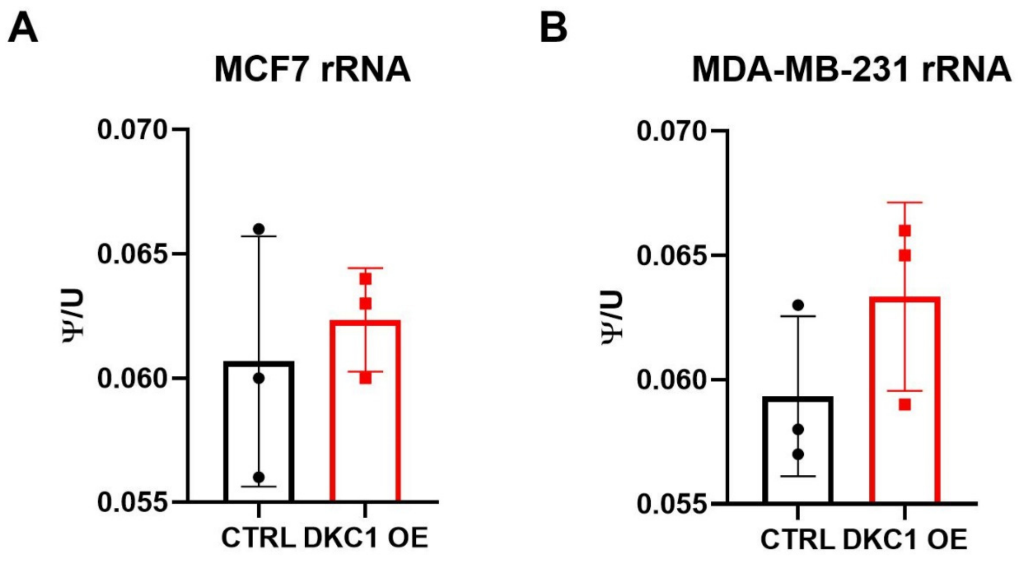

Figure S5. HPLC analyses of rRNA extracted from highly purified ribosomes from MCF7 and MDA-MB-231 DKC1 OE and CTRL cells. Results are shown as Ψ/U ratio. Two technical and three biological replicates for each experiment have been performed. No significant statistical differences have been found.

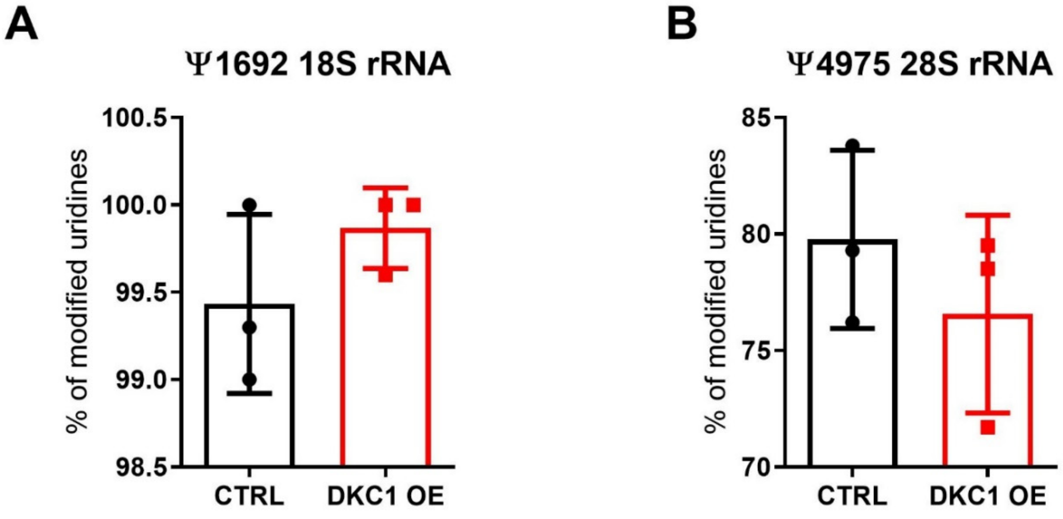

Figure S6. LC-MS analyses on Ψ1692 18S rRNA (**A**) and Ψ4975 28S rRNA (**B**) performed on MCF10A total cellular RNA. No significant differences in % of modified uridines were found after DKC1 overexpression. *N* = 3 biological replicates.

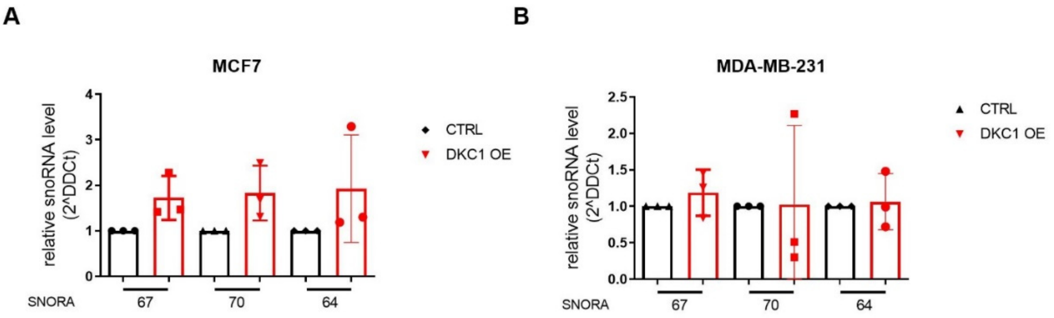

Figure S7. SNORA67, SNORA70 and SNORA64 expression evaluated by RT-qPCR in (**A**) MCF7 and (**B**) MDA-MB-231 stable dyskerin overexpression models. Experiments have been performed in technical triplicate of three biological replicates. No significant statistical differences have been found.

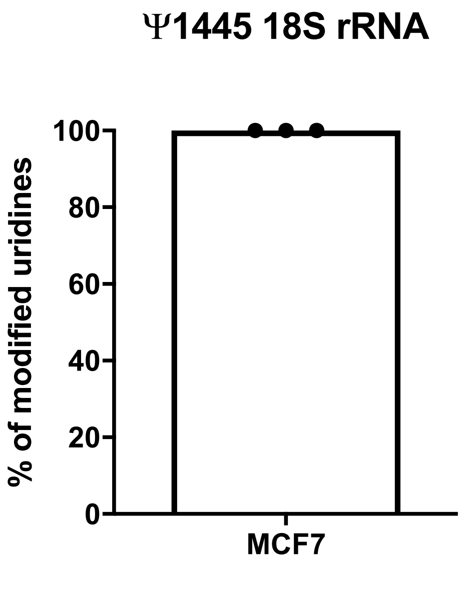

Figure S8. LC/MS analyses of Ψ1445 on 18S rRNA in parental MCF7 cell lines. Results have been obtained from three biological replicates. Results are shown as percentage of modified uridines and were calculated from the peak area.

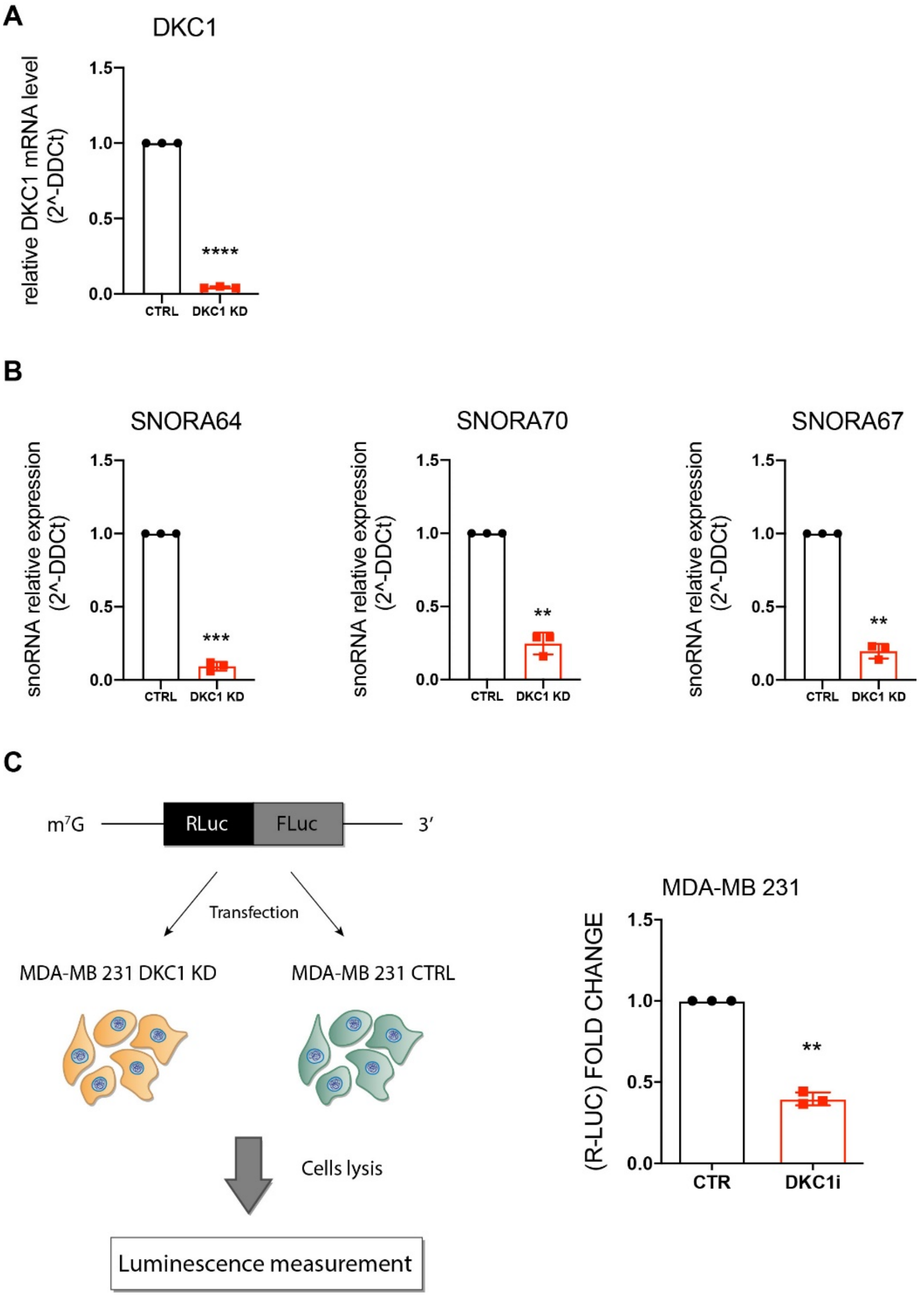

Figure S9. Evaluation of the transient DKC1 knockdown (KD) effects in MDA-MB-231 cells. (**A**) DKC1 mRNA expression evaluated by RT-qPCR. Results are shown as fold change respect to CTRL, set to (**B**) SNORA64, SNORA70 and SNORA67 expression evaluated by RT-qPCR in MDA-MB-231 transient dyskerin KD models. (**C**) mRNA translation efficiency assay on MDA-MB-231 DKC1 KD and control cells. All experiments have been performed in technical triplicate of three biological replicates. All data were analyzed by paired Student's t test, * $p < 0.05$; ** $p < 0.01$; *** $p < 0.001$, **** $p < 0.0001$. The error bars indicate the SD.

Supplementary Materials

Data Extraction from TCGA Dataset and Statistical Analyses

This study considers data available in the TCGA database and, specifically, the cohort named "Breast Invasive Carcinoma TCGA PanCancer" data [1]. The original data can be found here:

https://gdc.cancer.gov/about-data/publications/pancanatlas. Expression data are described as ″mRNA Expression, RSEM (Batch normalized from Illumina HiSeqRNASeqV2)″. cBioPortal is a standardized dataset named TCGA Pan-Cancer Clinical Data Resource (TCGA-CDR), which includes four major clinical outcome endpoints, including survival outcomes. We extracted data from the cBioPortal through the functions provided by the R package *cgdsr.*

Only ″Breast Invasive Lobular Carcinoma″ and ″Breast Invasive Ductal Carcinoma″ cohorts were taken into account and, after the exclusion of male subjects and subjects with missing data, the cohort counts n = 967 patients (mean age distribution 58 ± 13 SD). Survival analyses were performed and focused on the Overall Survival [2], considering a time range of 10 years, as for the primary breast cancer series shown in Figure 1A. For statistical analyses, patients were first divided into 3 groups based on quartiles of gene expression value (<1th quartile ″low″; >1th quartile and <3rd quartile ″medium″; >3rd quartile ″high″) and the association with a 10-year survival was tested using the log-rank test for trend (R package *survminer*).

Cell Culture and Transient DKC1 Overexpression

Human untransformed non tumorigenic HEK293FT cells and human osteosarcoma cell line U2OS were cultured in DMEM 4.5 g/L glucose supplemented with 10% FBS, 2 mM L-glutamine, 100 U/ml penicillin, 1 mg/ml streptomycin. Both cell lines were purchased from the American Type Culture Collection (ATCC) and cultured in a monolayer at 37 °C and 5% CO_2 in a humidified incubator.

To generate transient DKC1 overexpression cell models, we transfected both cell lines with 5 μg of both pCMV6-XL5-hDKC1 and relative control pCMV6-Entry plasmids (Origene - Figure S3), using Lipofectamine 2000 (Invitrogen) following manufacturer's protocol. Dyskerin overexpression has been evaluated by Western Blot analyses at 72, 96- and 120-h post-transfection (96 and 120 h not shown). Because of the best increase in dyskerin expression, 72 h have been chosen to perform all the experiments.

Transient DKC1 Silencing

For siRNA-mediated depletions, MDA-MB 231 cells were transfected with three specific siRNAs targeting DKC1 mRNA (Invitrogen, catalog number HSS102781: 5′-AACACCUGGAAGCAUAAUCUUGGCC-3′, HSS102782: 5′-UAAACAACCAGUCACCUUGGGAUCC-3′, HSS102785: 5′-GAAGUCACAACAGAGUGCAGGCAAA-3′) and an appropriate negative control (Cat. N° 12935300 - Invitrogen) using Lipofectamine RNAiMAX reagent (Invitrogen) in OptiMEM medium (Invitrogen) according to the manufacturer's instructions. Cells were harvested 72 h after the transfection.

References

1. Liu, J.; Lichtenberg, T.; Hoadley, K.A.; Poisson, L.M.; Lazar, A.J.; Cherniack, A.D.; Kovatich, A.J.; Benz, C.C.; Levine, D.A.; Lee, A.V.; et al. An Integrated TCGA Pan-Cancer Clinical Data Resource to Drive High-Quality Survival Outcome Analytics. *Cell* **2018**, 173, 400–416.e11, doi:10.1016/j.cell.2018.02.052.
2. NCI Dictionary of Cancer Terms. Available online: https://www.cancer.gov/publications/dictionaries/cancer-terms (accessed on 11th June 2020).
